# Left Bundle Branch Block Chest Pain Conundrum

**DOI:** 10.1155/2020/2724981

**Published:** 2020-02-19

**Authors:** Karthik Seetharam, Ayesha Cheema, Gary Friedman, Roman Pachulski

**Affiliations:** Division of Cardiology, St. John's Episcopal Hospital-South Shore, New York, New York, USA

## Abstract

Left bundle branch block is a pattern of altered ventricular depolarization and subsequently affects repolarization. These obscure patterns can affect the traditional ST segment shift criteria for the electrocardiographic detection of coronary insufficiency syndromes. Previously, patients with coronary ischemic pain and LBBB judged to be “new” (not previously documented) were considered to have ST elevation myocardial infarction (STEMI) warranting acute thrombolytic therapy. Current STEMI management favors emergent invasive angiography; however, recent data suggests the prevalence of coronary obstructive pathology may be as low as 50%. The application of more specific, less-sensitive Sgarbossa electrocardiographic criteria may reduce angiographic assessment in an otherwise high-risk population unlikely to tolerate further myocardial injury. We present a case that may facilitate a more nuanced EKG-based approach to distinguish those who may benefit from acute invasive angiography while reducing the frequency of unnecessary angiographic evaluation.

## 1. Introduction

**Figure 1:**
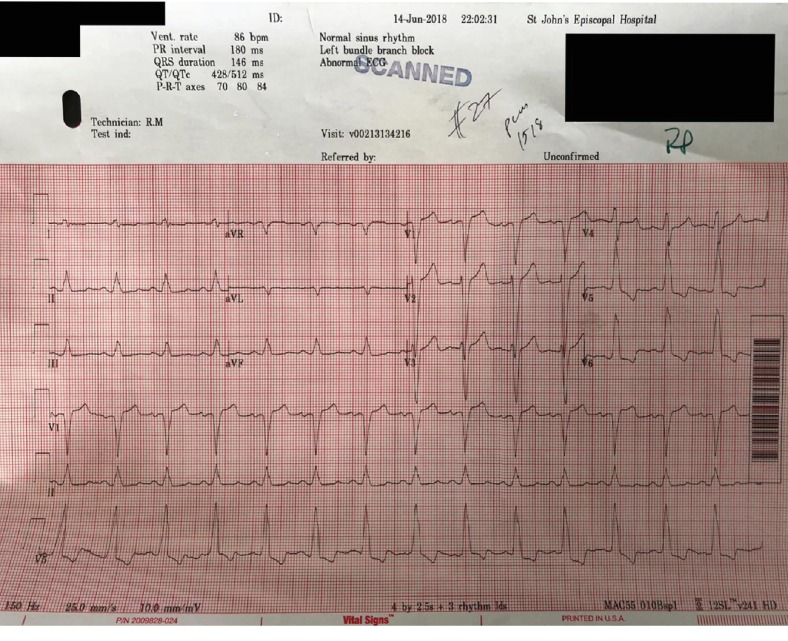
LBBB.

In the era of thrombolysis, all patients with left bundle branch block (LBBB) (Figure 1) were felt to warrant acute thrombolytic reperfusion [1]. Previous percutaneous coronary intervention (PCI) reports suggest obstructive pathology in LBBB patients may be as low as 50% [1] resulting in diminished enthusiasm for routine urgent catheterization. The primary goal is to rapidly submit to angiography, only those who have the highest likelihood of obstructive pathology while not withholding reperfusion from a population that likely has advanced cardiac disease. These patients are less likely to tolerate additional insult. We present a case that may facilitate a more nuanced acute reperfusion strategy for chest pain patients with resting QRS abnormalities that obscure the acute electrocardiogram (EKG) diagnosis of coronary obstruction.

## 2. Case History

**Figure 2:**
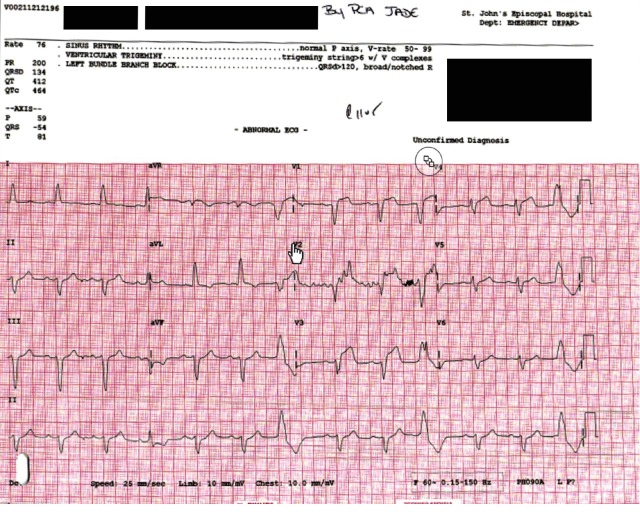
Patient EKG.

A 72-year-old male presented with exertional retrosternal chest pain, remote PCI, regional wall motion anomaly, and reduced left ventricular ejection fraction (LVEF) 29%. Initial troponin on presentation was 0.567 (*N* < 0.036). An electrocardiogram (EKG) (Figure 2) revealed QRS 122 ms, predominantly negative V1, but with terminal 80 ms negativity (S wave) in V6, and 3.5 mm horizontal ST elevation V1-V3. These findings were interpreted as left bundle branch block (LBBB) with insufficient ST shift by Sgarbossa [1] criteria, to infer acute coronary insufficiency, and the patient was treated conservatively with aspirin and sublingual nitroglycerin. After consultation by the cardiologists in our institution, this prompted a revised EKG diagnosis of left ventricular hypertrophy with QRS widening and STEMI (>2 mm ST elevation in 2 contiguous precordial leads). Treatment included dual antiplatelet therapy, heparin, and expeditious angiography. A proximal LAD 99% stenosis was successfully treated with drug eluting stent placement. On the following day, the peak troponin was 142. The patient did not experience clinical heart failure and recovered uneventfully.

## 3. Discussion

In this case, several clinical indicators suggested a high likelihood of acute coronary obstruction including prior PCI, regional wall motion anomaly, reduced LVEF, elevated troponin, and ischemic chest pain. All prior EKG's showed minimal (<130 ms) QRS widening with leftward terminal negativity as expected with prolonged intraventricular conduction incurred by LV hypertrophy rather than LBBB. As a result, all late forces are leftward and delayed by non-Purkinje (left bundle) fiber conduction.

While LBBB is generally a marker of advanced cardiac disease rendering further myocardial decline [1], low coronary artery disease prevalence may incur unnecessary invasive intervention and cost. Many automated EKG algorithms assign a diagnosis of LBBB to QRS > 120 ms, predominantly negative in V1 regardless of V6 morphology [2, 3]. This may misidentify up to 1/3 of patients with prolonged conduction due to left ventricular hypertrophy (chronic hypertension or valvular disease) as having LBBB [2]. Patients with left ventricular hypertrophy have prolonged, rather than disordered, depolarization and should not be disqualified from traditional EKG STEMI criteria. With true block of the left bundle, all late forces are leftward and cannot manifest terminal QRS negativity (S wave) in leads I, AVL, V5, and V6 [4]. Patients with QRS > 110 ms (rarely >130 ms) and terminal QRS negativity (S wave) in leads I, AVL, V5, and V6 are more accurately described as having left ventricular hypertrophy with QRS widening [5].

## 4. Conclusion

True block of the left bundle alters ventricular repolarization and invalidates traditional ST segment shift criteria for infarction. However, conduction prolongation due to left ventricular hypertrophy does not. The latter should be diagnosed when the QRS is <140 ms and there is terminal leftward negativity (S wave in I, AVL, V5, and V6). Prospective evaluation of a strategy where Sgarbossa criteria is only applied if QRS > 140 ms or QRS > 120 ms without terminal V6 negativity, while utilizing traditional criteria for those with intermediate QRS duration 120-140 ms with terminal V6 negativity to verify intervention accuracy may prove useful. In addition, the more accurate modified Sgarbossa criteria with a fractional rather than absolute ST displacement criterion could be evaluated.
